# Fast Trace Detection of Chlorpyrifos Vapors Using a Handheld Ion Mobility Spectrometer Operated near Ambient Temperature

**DOI:** 10.3390/toxics13100843

**Published:** 2025-10-02

**Authors:** Victor Bocoș-Bințințan, Ancuța-Maria Dodea, Tomáš Rozsypal, Adrian Pătruț, Gheorghe Roșian, Aurel-Vasile Martiniuc, Alin-Gabriel Moraru, Simina Vasc, Maria-Paula Bocoș-Bințințan

**Affiliations:** 1 Faculty of Environmental Science and Engineering, Babeș-Bolyai University, Str. Fântânele 30, 400294 Cluj-Napoca, Romania; anca.dodea@gmail.com (A.-M.D.); gheorghe.rosian@ubbcluj.ro (G.R.); alingabriel.moraru17@gmail.com (A.-G.M.); sdragomir199@gmail.com (S.V.); 2 SC Transcend SRL, Str. Arinilor 13, 400568 Cluj-Napoca, Romania; avmartiniuc@gmail.com; 3 Nuclear, Biological and Chemical Defence Institute, University of Defence, Vita Nejedleho 1, 68203 Vyskov, Czech Republic; tomas.rozsypal@unob.cz; 4 Faculty of Chemistry & Chemical Engineering, Babeș-Bolyai University, Str. Arany János 11, 400028 Cluj-Napoca, Romania; apatrut@gmail.com; 5 Raluca Ripan Institute for Research in Chemistry, Babeș-Bolyai University, Str. Fântânele 30, 400294 Cluj-Napoca, Romania; 6 Faculty of Veterinary Medicine, University of Agricultural Sciences and Veterinary Medicine, Calea Mănăştur 3-5, 400372 Cluj-Napoca, Romania

**Keywords:** chlorpyrifos, ion mobility spectrometry, trace detection, organophosphorus pesticides

## Abstract

Chlorpyrifos CPF (O,O-diethyl O-(3,5,6-trichloro-2-pyridyl) phosphorothioate), known also as Chlorpyrifos-ethyl, is one of the most utilized organophosphorus pesticides worldwide. Additionally, CPF could be used as a chemical warfare agent surrogate. Although its acute toxicity is not high, it is responsible for both a large number of intoxications and chronic, delayed neurological effects. In this work, it is reported for the first time the qualitative and quantitative response produced by CPF vapors, using a pocket-held Time-of-Flight Ion Mobility Spectrometer (ToF IMS) with a non-radioactive ionization source and ammonia doping, model LCD-3.2E (Smiths Detection Ltd.), operated near ambient temperature (below 30 °C). Spectra of CPF in positive ion mode included two distinct product ion peaks; thus, identification of CPF vapors by IMS relies on these peaks—the monomer M·NH_4_^+^ with reduced ion mobility K_0_ = ca. 1.76 cm^2^ V^−1^ s^−1^ and the dimer M_2_·NH_4_^+^ with K_0_ = ca. 1.47 cm^2^ V^−1^ s^−1^ (where M may be assignable to CPF molecule)—and positive reactant ions (Pos RIP) have K_0_ = ca. 2.25 cm^2^ V^−1^ s^−1^. Excellent sensitivity, with a limit of detection LOD of 0.72 ppb_v_ (10.5 μg m^−3^) and a limit of quantification LOQ of 2.41 ppb_v_ (35.1 μg m^−3^), has been noticed; linear response was up to 100 ppb_v_, while saturation occurs over ca. 1000 ppb_v_ (14.6 mg m^−3^). Our results demonstrate that this method provides a robust tool for both off-site and on-site detecting and quantifying CPF vapors at trace levels, which has strong implications for either industrial hygiene or forensic investigations concerning the pesticide Chlorpyrifos, as well as for monitoring of environmental contamination by organophosphorus pesticides.

## 1. Introduction

Chlorpyrifos (CPF), known also as Chlorpyrifos-ethyl, is an organophosphorus (OP) insecticide with large spectrum, widely used against a variety of pests (mainly insects, but also worms, larvae, fleas, locusts—in both outdoor and indoor applications) and is considered as one of the most used insecticides in agriculture both in the U.S. and in Europe [1,2]. CPF was introduced in 1965 by Dow Chemical Company (Dow AgroSciences), then manufactured and marketed in huge amounts (thousands of tons per year), especially under the tradenames Dursban™ and Lorsban™.

Organophosphorus (OP) compounds induce acute neurotoxic effects by inhibiting the enzyme acetylcholinesterase in neurons’ synapses; this way, cholinergic hyperstimulation is caused. Main symptoms of acute intoxications with OP compounds (either pesticides or nerve chemical warfare agents) are miosis, salivation, lacrimation, urination and defecation, then muscle twitching, convulsions and, as ultimate effect, death [3]. Infants and children are particularly vulnerable to chronic adverse effects generated by OP pesticides; thus, their exposure to OP pesticides (in utero and/or post-birth) has been associated with issues related to neuro-development, decreasing the normal gestation period and alteration of fetal growth [4]. Poisoning with CPF may also affect, besides the central nervous system, the cardiovascular and respiratory systems. While CPF is far less toxic than organophosphorus nerve chemical warfare agents, it shares similar toxicological effects, making it, therefore, a useful tool for research and training related to nerve chemical weapons exposure.

There are also many adverse eco-toxicological effects related to Chlorpyrifos, for instance, toxicity towards pollinating insects, birds, fish and other aquatic organisms, organisms living in soils and domestic animals. Like most OPs, CPF has, fortunately, a relatively short half-life inside biological tissues; for instance, a single exposure to CPF results in ca. 24 h residence time in blood and ca. 60 h in fat. Also, CPF does not have a high potential for bioaccumulation in mammals. The half-life of CPF in indoor environments has been estimated to be approximately one month [5]. However, investigations concerning the treatment with CPF demonstrated that it was still present up to 8 years post application; CPF has persisted into/onto various objects and surfaces (such as toys, pavements or furniture) for up to two weeks after its indoor application [5].

The most relevant physico–chemical and toxicological properties of Chlorpyrifos are summarized in Table 1.

It is important to emphasize that Chlorpyrifos is sensitive to light, moisture, various alkaline compounds (e.g., bleach) and also to microbial degradation. The half-life of Chlorpyrifos in water is pretty short—from several days to a couple of weeks. CPF may be adsorbed to sediments and organic matter; thus, its half-life in soil may usually be 2 to 4 months, but it may extend to over one year. The granular commercial formulations of CPF are found to be even more persistent (up to 6 months). The major biological metabolite and environmental breakdown product of CPF hydrolysis is 3,5,6-trichloro-2-pyridinol (TCP: C_5_H_2_Cl_3_NO), accompanied by either diethyl phosphorothioate (DETP: C_4_H_11_O_3_PS) or diethyl phosphate (DEP: C_4_H_11_O_3_PO) [5,6].

Detection of various OP compounds present as vapors in air samples can be realized by using a diversity of analytical techniques, from simple colorimetric techniques and photoionization detectors (PIDs) to more complex instruments such as gas chromatographs coupled often with mass spectrometers (GC-MSs), flame photometry detectors (FPDs), or surface acoustic wave (SAW) sensors [7]. For most toxic OP compounds—like the nerve chemical warfare agents—the preferred analytical technology used on-field nowadays is ion mobility spectrometry (IMS) [8].

Ion mobility spectrometry (IMS) has been continuously building an increasing recognition during the last decades and has become a mature analytical technology. IMS possesses extreme versatility, being perfectly suited for trace detection and identification of many classes of chemicals—either present in air samples as vapors, or vaporized from liquid and solid samples—after their soft ionization at atmospheric pressure [8]. IMS possesses a superb sensitivity, often presenting detection limits of low ppb_v_ for a great variety of compounds, with no pre-concentration. The high speed of ion separation, which takes place at atmospheric pressure and in the gaseous phase, can also be regarded as the main operational advantage of IMS; since a single ion mobility spectrum is obtained in 20 milliseconds, the whole analysis run usually takes just several seconds. IMS instrumentation is pretty simple, which has translated towards highly compact, hand- and even pocket-held rugged IMS devices. The most important fields of IMS applicability are detection and identification of all types of chemical weapons [9], fast sensing of explosives (including acetone-based homemade ones) [10,11], illegal drugs plus their associated precursors [12,13] and a wide range of toxic industrial chemicals [14,15]. IMS technology was also successfully used in other domains—forensic approaches, various industries for quality control and quality assurance, toxicology, bio-medicine, environmental protection and monitoring or even space research [8].

IMS relies on two main stages. The first and crucial one is the ionization of neutral compounds, occurring at atmospheric pressure and in the gas phase. The second stage is the ions’ separation in a neutral drift gas, under the influence of a direct-current electric field with relatively low intensity (E < 500 V/cm). Nowadays, this IMS analytical technique is named the Drift Tube (DT) IMS, or Time-of-Flight (ToF) IMS. Therefore, in ToF IMS, the created ions are separated in time by the longitudinally applied electrical field, while the analytical output is a practical, very fast chromatogram. In ToF IMS, separation of different ions occurs because they possess different speeds and mobilities when traveling against a flow of neutral drift gas (recirculated purified air in portable IMS instruments). The “motor” of ions’ displacement is the longitudinal d.c. electric field applied onto the IMS cell. Formation of ions in IMS is a fast but complex process, involving two successive steps—firstly, the generation of primary (reactant) ions starting from molecules present in the drift gas (usually purified air), and secondly, the formation of secondary (product) ions, which will include the entire molecule of the target analyte. Therefore, when an ionization source is used in air, a series of fast and complex ion–molecule collisional processes will produce the initial batch of reactant ions. For instance, in positive ion operation mode, with a radioactive ionization source and by using water vapor chemistry, the positive reactant ions formed will be predominantly hydrated protons (H_2_O)_x_H^+^, accompanied by (H_2_O)_y_NH_4_^+^ and (H_2_O)_z_NO^+^, while in negative ion mode, the dominant negative reactant ions will be the (H_2_O)_n_O_2_^−^ clusters. After reactant ions are generated, if the target analyte has a proton affinity (or electron affinity in the negative ion mode) higher than that of the reactant ions, the latter can transfer their electrical charge to the molecules of the analyte, and this leads to the formation of product ions that include the whole analyte molecule. The concentration level of water vapor present in the IMS cell is paramount and must therefore be strictly controlled and kept at a low level, since water vapor has a crucial role in the whole ion–molecule chemistry that occurs at atmospheric pressure [14]; otherwise, the IMS analytical performance will degrade significantly.

The constant that links the drift speed v_d_ of an ion to the electric field intensity E that propels that ion through the IMS cell is called “ion mobility” K, according to the following equation: v_d_ = K·E = l_d_/t_d_ (where l_d_ = drift length of the IMS cell and t_d_ is the drift time of an ion). Of course, one may further infer that K = v_d_/E = l_d_/(E·t_d_). By normalizing ion mobility for both pressure and temperature inside the IMS measurement cell one shall obtain the so-called “reduced ion mobility K_0_”, a parameter that is used in a consistent manner for accomplishing qualitative characterization of the compound that generates that/those product ion(s): K_0_ = K·(T_ambient_/T_cell_)·(P_cell_/P_atmospheric_) [8].

The main aim of the present paper is to demonstrate the fast detection and quantification of vapors of Chlorpyrifos present at ultra-trace levels (<1 ppm_v_) in air samples by employing a handheld ToF IMS instrument with corona discharge ionization and ammonia doping, operated near ambient temperature. Both the risk of acute/chronic intoxications to humans and other potential ecotoxicological effects of Chlorpyrifos fully justify the need for fast trace detection of its vapors using the rapid analytical technique of IMS. Even if the commercial use of CPF has been restricted since 2000, this necessity remains stringent. The literature on the detection of CPF by IMS is relatively scarce, especially as it approaches solid and liquid matrices, as discussed later.

Our intention was to demonstrate, through this study, the qualitative and quantitative capabilities of portable IMS devices with corona discharge non-radioactive ionization operated near ambient temperature for the fast sensing of CPF vapors. Evaluating the performance of such handheld portable IMS devices constitutes an important novelty and a strong point of the paper, especially since previous studies have employed only bulky stationary desktop IMS instruments or even more complex desktop hyphenated GC–IMS systems. The application of portable IMS technology for rapid on-site detection of CPF vapors was highlighted. Our research is based on preparing a series of standard atmospheres with known concentrations of CPF vapors, in the ultra-trace (under 1 ppm_v_) range.

A large number of studies have approached the analysis of CPF in complex environmental matrices, and we comprehensively and critically reviewed them; all these studies provide valuable insight into CPF analysis in various environmental (water, fruits and vegetables) and particulate samples. However, to the best of our knowledge, none of the previous reports have investigated CPF vapors using a handheld IMS instrument operated near ambient temperature, as all prior IMS studies employed stationary instruments operating at high temperatures (150–240 °C). Our current work is therefore intended to significantly complement the existing literature by using a portable IMS device suitable for real-time CPF vapor sensing at relatively low (ambient) temperature.

## 2. Materials and Methods

### 2.1. The IMS Instrument

For detection and quantification of CPF vapors, a highly portable, pocket-sized commercial ToF IMS instrument (size ca. 18.0 × 11.5 × 4.5 cm and weight of ca. 0.6 kg), model LCD-3.2E (Lightweight Chemical Detector), manufactured by the company Smiths Detection Ltd., Watford, UK, was used. The instrument is built around a twin-design miniature IMS cell, which means that two IMS cells are used simultaneously. Both cells use a classical design consisting of alternating conducting elements placed onto an insulating cylinder, and each has a drift length of ca. 3 cm; the intensity of d.c. electric field E is ca. 270 V cm^−1^. On the basis of this “twin cell” configuration, this IMS platform generates both positive and negative ion mobility spectra at the same time, which is a consistent advantage. The LCD-3.2E is operated near ambient temperature, at ca. 301 K (28 °C), while the pressure inside the measurement cells is atmospheric pressure, of ca. 1000 mbar. A non-radioactive ionization source, based on a point-to-plane corona discharge, was the one employed in the LCD-3.2E system. For a series of reasons, a source based on corona discharge is a more viable ionization alternative to the almost universally used radioactive sources based on beta isotopes (mainly Ni-63, or more rarely tritium H-3); one major advantage to quote here is the lack of any legal and bureaucratic burdens associated with manipulating, using or transporting any radioactive material. Besides that, the ionization sources with corona discharge generate a higher amount of reactant ions (hence a higher signal) compared to radioactive sources, while their ionization chemistry is quite similar to that of radioactive sources. The LCD-3.2E uses dry air as drift gas, which is continuously recirculated through a filter based on molecular sieve. Also worth mentioning is that the same purifying filter delivers, in a continuous manner, low amounts of gaseous ammonia, which is the dopant that seriously improves selectivity in positive ion mode. To the best of our knowledge, this model of IMS instrumentation is maybe the most compact and miniaturized commercial ToF IMS system that exists currently on the market and is perfectly capable of sensing and identifying rapidly, in a matter of seconds, both gases and vapors belonging to the TIC and CWA categories. The LCD-3.2E instrument has already been described in reference [15], including its schematic diagram.

This IMS system was connected, through the proprietary software TrimScan2, ver. 0.4.0 (Smiths Detection Ltd., UK), to a PC. This way, all resulting experimental data were saved as ion mobility spectra (positive and negative) on the hard drive of the PC; then, the spectra were exported to MS Excel files.

If ammonia is being used as a dopant in IMS, then the major positive reactant ions shift from hydrated proton clusters (H_2_O)_n_H^+^ towards ammonia-hydrated ones, having the general formula (H_2_O)_m_NH_4_^+^. When a small volume of air sample with CPF vapors is sent into the IMS cell, the non-radioactive ionization source using the corona discharge ionizes the molecules of CPF in a soft manner and eventually generates product ions that include the molecule of the target analyte. All formed ions—both reactant and product—are injected inside the drift (separation) region by the opening of the shutter grid, then they “fly” through the distance from the shutter grid to the detector (the drift length) and eventually attain a drift speed v_d_ that has a constant value of several meters per second. When a specific ion (either reactant or product ions) arrives at the detector, a very low ion current, usually in the range of tens of pA, is produced, then amplified and measured. As a consequence, every compound will have its own characteristic drift speed when traveling through the neutral drift gas (air at atmospheric pressure), and for this reason, its drift time can be utilized for its identification, similar to the retention time in chromatography.

### 2.2. Reagents, Sampling and Work Flow Procedure

A liquid standard reference material (SRM) containing CPF in hexane, with a concentration of 2 mg L^−1^ (Dr. Ehrenstorfer GmbH, Augsburg, Germany), was used in order to create test atmospheres that have very low concentrations of CPF vapors in air. These standard atmospheres were produced by injecting known volumes of standard solution inside 0.72 L glass jars with screw caps; a series of microliter GC syringes with volumes of 5, 10, 20, 50, 100 and 500 μL were used for this purpose.

Glass jars were stored in a heating oven for 30 min, at a temperature of ca. 50 °C, in order to allow vaporization of CPF. Between the jar’s opening and metallic screw cap, a triple sheet of 10 micron-thick aluminum foil was inserted; after the equilibration period, the cap was removed, then a 1 cm hole was made in the aluminum layers, and the analytical sampling nozzle of the IMS spectrometer was inserted in order to sample.

Therefore, in order to perform the necessary IMS measurements, the standard atmospheres with known, low concentrations of CPF vapors (<1 ppm_v_) were produced by using a static method. Using the described static method, firstly, a series of six different test atmospheres containing very low concentrations (≤50 ppb_v_) of CPF vapors were generated: 2.5 ppb_v_, 5 ppb_v_, 10 ppb_v_, 20 ppb_v_, 40 ppb_v_ and 100 ppb_v_, respectively.

Higher concentrations of CPF vapors, up to ca. 1000 ppb_v_, were obtained using a commercial pesticide formulation called Nurelle-D (manufactured by Agriphar S.A., Ougree, Belgium), which is an emulsified liquid concentrate containing 500 g CPF L^−1^. The same experimental procedure was used as for low concentrations, after repeatedly diluting the Nurelle-D with hexane in order to decrease the CPF concentration in the standard solution to 0.5 mg cm^−3^.

Table 2 presents succinctly the experimental conditions used for generating all the above-mentioned standard/test atmospheres.

## 3. Results

The experimental data obtained from the IMS model LCD-3.2E (ion mobility spectra, in positive ion mode) were recorded step by step, from the lowest concentration to the highest one, for every trace concentration of vapors of CPF obtained in the afferent static test atmosphere. IMS measurements for any concentration level were performed in triplicate; standard deviations between 5% and 7% were observed.

The results of our research are shown synthetically in Table 3, where C_CPF_ is the concentration of CPF vapors in the standard atmosphere (see Table 2).

**Figure 1:**
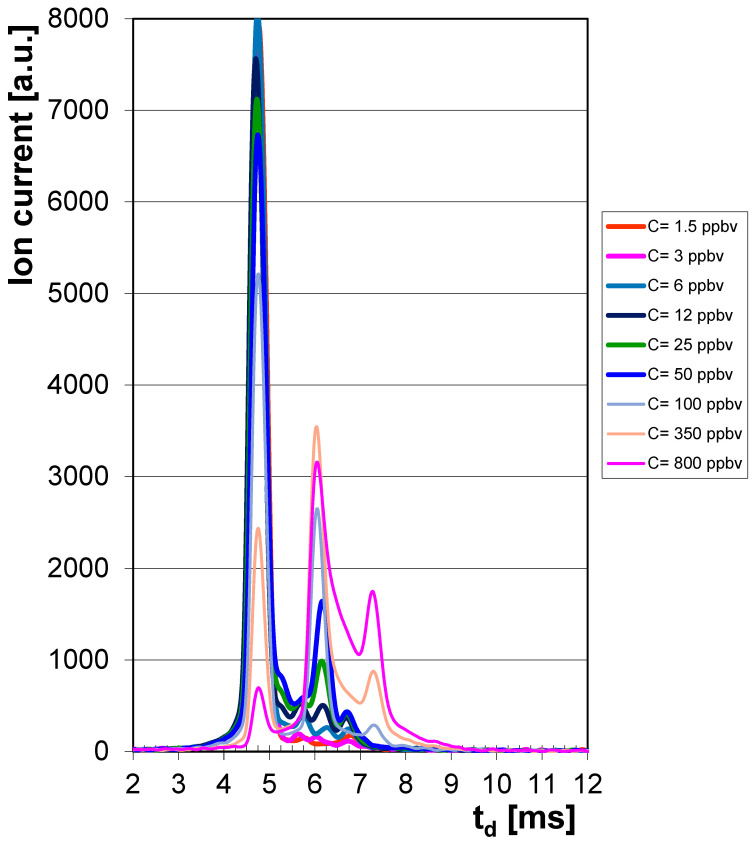
Ion mobility spectra from CPF, obtained in the positive ion mode, for vapor concentrations between 1.5 and 800 ppb_v_. Positive RIP (reactant ion peak) is the first peak in the spectrum, at t_d_ = ca. 4.7 ms, then the monomer product ion peak is the second feature (at t_d_ = ca. 6.1 ms) and finally the dimer product ion peak from CPF is the third feature in the spectrum, at t_d_ = ca. 7.3 ms. The scale of drift time has been narrowed, from 2 to 12 milliseconds, for reasons of clarity.

For low CPF vapor concentrations (≤50 ppb_v_), ion mobility spectra showed only one product ion peak (PIP) in the positive ion mode, observed at a drift time t_d_ = ca. 6.1 ms and assignable to the monomer product ion, with probable structure CPF·NH_4_^+^. At higher CPF concentrations (≥100 ppbv), a dimer product ion (assignable to CPF_2_·NH_4_^+^) appeared at a drift time t_d_ = ca. 7.3 ms. The peak of positive reactant ions (Pos RIP) was noticed to appear at a drift time t_d_ = ca. 4.7 ms. All positive ion mode IMS spectra for low vapor concentrations of CPF (from 1.5 to 800 ppb_v_) are presented in Figure 1; spectra clearly illustrate electrical charge conservation principle—the intensity of the product ion peaks produced by CPF increases when vapor concentration of CPF increases, and, consequently, at the same time, the intensity of the positive reactant ion peak declines.

The appearance of proton-bound dimer product ions is observed for several classes of compounds, such as ketones or organophosphorus compounds, and typically occurs when the analyte concentration exceeds a certain threshold [8]. The phenomenon results, therefore, from the enrichment of vapor-phase analyte molecules at higher concentrations, correlated with the life of the dimer product ion long enough for this ionic species to reach the detector and hence to generate an ion current.

CPF did not produce an IMS response (spectrum) in the negative operation mode.

Because n-hexane used as a solvent for CPF possesses a proton affinity, PA, far lower than that of ammonia (the PA of hexane is ca. 676 kJ mol^−1^, while PA of ammonia dopant is ca. 854 kJ mol^−1^), molecules of n-hexane will not become ionized by collisional charge transfer with ammonia-based positive reactant ions and hence they will not generate any peak in the positive ion mode IMS spectrum.

**Figure 2:**
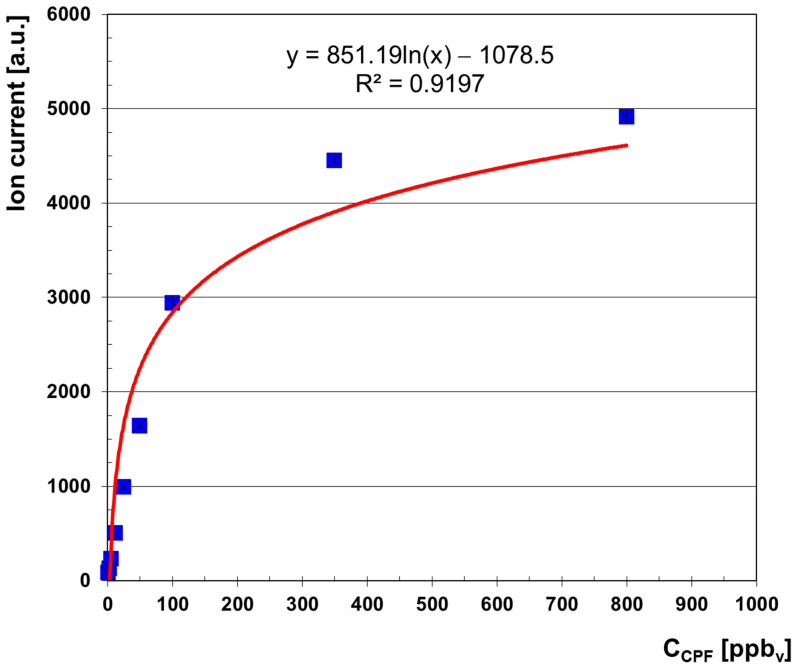
Calibration for CPF, in the positive ion mode.

Quantitative information (peak intensities) has been utilized to build the afferent calibration curve and then to evaluate the quantitative response offered by the LCD-3.2E IMS instrument to vapors of CPF. All nine concentrations of CPF vapors were employed—the six very low concentrations + the three larger concentrations. This calibration curve is presented in Figure 2; the ion current (analytical signal) is considered as the sum of the ion current of the monomer product ion and that of the dimer product ion. A logarithmic-type trend line equation has also been included.

IMS spectra contain the qualitative information (that includes the drift time t_d_ of a specific ion and its reduced mobility K_0_) and also the quantitative information that resides in peak height. Drift time is relatively proportional to the ion’s mass and its size and inversely proportional to the ion’s electrical charge. Summarizing and simplifying, any ion mobility peak in the spectrum can be characterized by using three numbers: (1) drift time t_d_ (in ms); (2) reduced ion mobility K_0_ (in cm^2^ V^−1^ s^−1^); and (3) peak height h_max_ (in a.u.).

In Table 4, the relevant qualitative information is presented, which includes both drift times and the associated reduced ion mobilities for a positive reactant ion peak and, of course, for product ion peaks of CPF (both monomer and dimer). A common feature of OP compounds is the formation, when vapor concentration increases, of a pair of two product ion peaks [8,9]—a monomer and a dimer—and CPF does not make an exception.

Reduced ion mobility K_0_ has been checked and re-calculated using the accepted mobility standard compound 2,4-lutidine (2,4-dimethylpyridine), from which the “IMS cell constant” has been calculated. This method has the consistent advantage of considering all possible inhomogeneity of the electric field E that propels the ions inside the IMS measurement cell. Moreover, by using this cell constant in calculating K_0_, the need for highly accurate measurements for relevant instrumental parameters (electric field intensity E and drift length l_d_) and environmental ones (pressure and temperature inside the IMS cell) is eliminated. The compound 2,4-lutidine has already been largely accepted as a chemical standard for mobility in the positive ion mode of operation, with the following known reduced ion mobilities: K_0 of standard (Lutidine monomer)_ = 1.95 cm^2^ V^−1^ s^−1^ and K_0 of standard (Lutidine dimer)_ = 1.43 cm^2^ V^−1^ s^−1^, respectively [16]. The constant of the ion mobility cell (noted here with A) is, in fact, nothing but the product between the reduced mobility K_0_ of the chemical standard and the drift time t_d_ of the product ion generated by that chemical standard. Consequently, the following simple equation is used in order to find the reduced ion mobility of peaks associated with CPF·vapors: A = K_0 of standard (Lutidine dimer)_·t_d of standard (Lutidine dimer)_ = K_0 of analyte (CPF)_·t_d of analyte (CPF)_. Since the measured t_d of standard (Lutidine dimer)_ = 7.46 ms, then the cell constant of the IMS instrument will be calculated as follows: A = 1.43 cm^2^ V^−1^ s^−1^·7.46·10^−3^ s = 10.668·10^−3^ cm^2^ V^−1^.

The normalization of drift time of both product ions (monomer and dimer) against the drift time of positive reactant ion peak (Pos RIP) is also of interest; thus, the ratio between the drift times of PIPs and Pos RIP is as follows: t_d PIP 1_/t_d Pos RIP_ = K_0 Pos RIP_/K_0 PIP1_ = 1.278 for the monomer product ion CPF·NH_4_^+^, and t_d PIP 2_/t_d Pos RIP_ = K_0 Pos RIP_/K_0 PIP2_ = 1.536 for the dimer product ion CPF_2_·NH_4_^+^, respectively.

Resolving power of the IMS instrument model LCD-3.2E, R_IMS_, is the ratio between the drift time of an ion and its width at half height (R_IMS_ = t_d_/Δt_d_) and has been determined for all peaks existing in the ion mobility spectrum; the found results are given in Table 5. These resolving power-calculated values, which ranged between 13.5 and 17.3, are perfectly normal for handheld commercial instruments with short IMS measurement cells, like the case of the LCD-3.2E device.

### Validation

Validation has been performed by using a simple procedure to quickly assess the suitability of the IMS-based analytical method employed. Thus, the evaluated parameters of the proposed method using IMS were as follows: limit of detection, limit of quantitation, sensitivity, linear response range, and accuracy.

Limit of detection (LOD) is the lowest concentration that produces a signal-to-noise (S/N) ratio equal to 3, and limit of quantification (LOQ) is defined as the lowest concentration that generates an S/N ratio of 10. Sensitivity S is the change in signal Y (peak height) that appears when the analyte’s concentration is incrementally changed (S = ΔY/ΔC). The background signal—described as the average of the background noise—has been calculated by using the latest 500 data points (those output ion currents obtained for drift times ranging from 10.00 to 20.00 ms, with increments of 0.02 milliseconds) for the blank IMS spectra; this average value of the signal (ion current) in positive ion operation mode has been found to be of ca. 8 a.u. The relevant figures of merit related to quantification of CPF vapors (LOD, LOQ, linear range and sensitivity S), which were determined based on the IMS response over the linear range (≤50 ppb_v_ CPF), are as follows:

- Limit of detection LOD = 0.72 ppb_v_
- Limit of quantification LOQ = 2.41 ppb_v_
- Linear range: 2.41—50 ppb_v_
- Equation of linear regression: Y = 33.155·X + 49.622 (R^2^ = 0.9893)
- Sensitivity S = 32.1 a.u./ppb_v_.

Precision has been evaluated using analyses in triplicate (see Table 3). Accuracy was assessed by using relative standard deviation, RSD (also known as coefficient of variation, CV), which was determined to be from 5% to 7% for the product ion peaks, PIPs. The repeatability of results was good, since RSD was less than 10%.

## 4. Discussion

The logarithmic-type allure of the built calibration curve (Figure 2) is characteristic for any IMS-type response [8]. There is also a good resemblance between the quantitative IMS response provided by the portable ToF IMS device equipped with a non-radioactive (corona discharge) ionization source that has been used by us (the LCD-3.2E) and that generated by other ToF IMS systems that rely on radioactive ionization sources.

IMS spectra obtained for the highest vapor concentration of CPF of 800 ppb_v_ indicate that the saturation threshold was almost reached, since at this concentration level, the reactant ions peak was still present but had a much-diminished intensity of just about 10% of its initial height. Total saturation means that the whole amount of reactant ions has been depleted; its consequence is the total disappearance of the peak of reactant ions from the IMS spectrum. It must be strongly emphasized that saturation must be avoided, since it will lead to the persistent contamination of both the IMS cell and all the internal surfaces that came in contact with the vapors of the analyte; in other words, this type of contamination generates totally unwanted false alarms and memory effects.

Potential interferences from other chemicals are always a potential issue, and this may also happen in the case of IMS instruments. On the other hand, because CPF produces, at trace levels of just hundreds of ppb_v_, two peaks in the positive ion mode simultaneously, the qualitative identification of CPF using two time windows (monomer + dimer product ions) is clearly much more reliable, as compared to that using a single product ion peak. Common solvents and the majority of other vapors are very unlikely to form positive product ions having exactly the same drift times simultaneously (and reduced ion mobilities) as the two product ions generated by CPF, as most of these compounds have a proton affinity lower than that of ammonia-based reactant ions (854 kJ mol^−1^). Therefore, significant interferences from usual solvents under our experimental conditions are not anticipated at all. This enhanced chemical selectivity in positive ion mode is a crucial advantage that occurs from modifying chemical ionization processes at atmospheric pressure when using dopants such as ammonia.

Reduced ion mobilities for the monomer and dimer product ion peaks generated by Chlorpyrifos (CPF) vapors in the positive mode have been determined as K_0_ = ca. 1.76 cm^2^ V^−1^ s^−1^ for the monomer (assumed to be CPF·NH_4_^+^) and K_0_ = ca. 1.47 cm^2^ V^−1^ s^−1^ for the dimer (assumed to be CPF_2_·NH_4_^+^), using 2,4-lutidine as mobility standard.

By examining the quantitative response (Figure 2) and all ion mobility spectra obtained for the whole range of CPF vapor concentrations (Figure 1), one may conclude the following:

1. Minimum measured concentration of CPF vapors was 1.5 ppb_v_ (0.022 mg m^−3^).
2. Linear dynamic range is from 2.4 ppb_v_ (0.035 mg m^−3^) to ca. 100 ppb_v_ (1.46 mg m^−3^) CPF.
3. Saturation is estimated to appear at >1000 ppb_v_ (14.57 mg m^−3^) CPF.

The IMS responses for CPF were all obtained in the positive ion mode, which strongly suggests that CPF has a proton affinity (PA) higher than that of ammonium positive reactant ions of 854 kJ mol^−1^. The pocket-held IMS instrument model LCD-3.2E responded quickly, in real time (several seconds only), to vapors of target analyte CPF.

A comprehensive literature survey about the detection and quantification of CPF using various IMS instruments is summarized in Table 6. One may rapidly observe that almost all references have provided information regarding CPF extraction from various environmental samples (water, fruits and vegetables) with subsequent detection by IMS.

Information concerning detection and quantification of CPF using DT-IMS, summarized in Table 6, reveals a series of conclusions: there is a large packet of 21 papers (ref. [17,18,19,20,21,22,23,24,25,26,27,28,29,30,31,32,33,34,35,36,37]) by several groups of Iranian researchers, which, in fact, heavily focus on their efforts towards analyzing CPF present in various liquid and solid samples (water, fruits and vegetables), using a variety of elaborated and time-consuming methods of extraction and pre-concentration, most of them relying on micro-extraction. However, an objective but critical examination of all these papers clearly indicates that the information related to IMS detection is relatively poor and/or incomplete, since the authors only included the IMS spectrum, but they did not provide even the exact drift time of the CPF peak; it has to be mentioned that drift times included in Table 6 for ref. [17,18,19,20,21,22,23,24,25,26,27,28,29,30,31,32,33,34,35,36,37] were estimated visually by us from the spectra included in those papers. The reduced ion mobility K_0_ of the CPF product ion was also not indicated, with the exception of two papers that have reported the K_0_ of CPF using nicotinamide as mobility standard—ref. [30], with K_0_ = 1.37 cm^2^ V^−1^ s^−1^ and ref. [32], with K_0_ = 1.27 cm^2^ V^−1^ s^−1^. All these papers ([17,18,19,20,21,22,23,24,25,26,27,28,29,30,31,32,33,34,35,36,37]) claim to have detected CPF in the positive ion mode and at high temperatures of the IMS cell, between 150 and 235 °C, but none of them accomplished the net identification of CPF using a mass spectrum (e.g., by employing the tandem GC-MS), which is another potential drawback. Another common finding is the presence of just one product ion peak generated by CPF, although in the IMS spectrum from ref. [35,36], a second small peak emerges. Even if the authors of previous studies have not provided the reduced ion mobilities for the product ion assigned to CPF, based on their included information regarding the IMS instruments used (drift length l_d_; temperature of the IMS cell T_IMS_, pressure inside the IMS cell P_IMS_—assumed it as 760 Torr everywhere—and intensity of the d.c. electric field E, respectively), the K_0_ was calculated by us in order to compare these values with our findings. In this regard, the calculated reduced ion mobilities are as follows: K_0_ = 1.180 cm^2^ V^−1^ s^−1^ for [17]; K_0_ = 1.133 cm^2^ V^−1^ s^−1^ for [18]; K_0_ = 1.086 cm^2^ V^−1^ s^−1^ for [19]; K_0_ = 1.232 cm^2^ V^−1^ s^−1^ for [20]; K_0_ = 1.395 cm^2^ V^−1^ s^−1^ for [21]; K_0_ = 1.414 cm^2^ V^−1^ s^−1^ for [22]; K_0_ = 1.332 cm^2^ V^−1^ s^−1^ for [23]; K_0_ = 0.913 cm^2^ V^−1^ s^−1^ for [24]; K_0_ = 1.521 cm^2^ V^−1^ s^−1^ for [25]; K_0_ = 1.380 cm^2^ V^−1^ s^−1^ for [26]; K_0_ = 1.076 cm^2^ V^−1^ s^−1^ for [27]; K_0_ = 0.847 cm^2^ V^−1^ s^−1^ for [28]; K_0_ = 1.675 cm^2^ V^−1^ s^−1^ for [31]; K_0_ = 2.098 cm^2^ V^−1^ s^−1^ for [33]; K_0_ = 0.907 cm^2^ V^−1^ s^−1^ for [34]; K_0_ = 1.251 cm^2^ V^−1^ s^−1^ for [35]; K_0_ = 1.257 cm^2^ V^−1^ s^−1^ for [36] and K_0_ = 1.204 cm^2^ V^−1^ s^−1^ for [37]. One easily observes that calculated reduced ion mobilities K_0_ range from 0.847 to 2.098 cm^2^ V^−1^ s^−1^, so there is an interval of about 1.25 cm^2^ V^−1^ s^−1^ for K_0_ values, which is not reasonable. The only two papers that reported K_0_ based on using nicotinamide as mobility standard (of 1.37 [30] and 1.27 cm^2^ V^−1^ s^−1^ [32], respectively) seem, however, to be the most reliable in terms of K_0_ determination; these reduced mobilities are the closest to our found value for the dimer product ion of CPF (ca. 1.47 cm^2^ V^−1^ s^−1^). In other words, it is very possible that the unique peak described by the Iranian researchers is in fact the dimer product ion peak of CPF.

Apart of this avalanche of papers written by Iranian scientists, only one distinct article dealing with CPF detection using DT (ToF) IMS was found; this particular work has also been performed using a commercial desktop IMS instrument with radioactive ionization source and operated at high temperature; oddly enough, the authors report that CPF produced an IMS response in the negative ion mode, with just one product ion peak having a reduced ion mobility K_0_ = 1.56 cm^2^ V^−1^ s^−1^ [38].

Therefore, based on our rather exhaustive literature survey (Table 6), it seems that the current approach of sensing CPF vapors using a handheld DT IMS instrument with a corona discharge ionization source and operating near ambient temperature is the first one described in the literature. As mentioned already, all papers dealing so far with IMS detection of Chlorpyrifos [17,18,19,20,21,22,23,24,25,26,27,28,29,30,31,32,33,34,35,36,37,38] accomplish the IMS analysis at high temperatures (from 150 °C to ca. 240 °C), while the analyzed samples with CPF were either liquids (in most cases) or solid particles.

Assigning the identity of ions (both reactant and product) generated inside the IMS cell is possible with a high degree of reliability only by coupling the IMS with a mass spectrometer, which performs the identification of the ions formed in the IMS instrument. The hyphenated IMS-MS systems were utilized, especially with the goal of revealing the real identity of ions [14]. Assigning the identity of ions produced by CPF vapors inside the IMS cell with LCD-3.2E was therefore not intended and also not feasible in this study. On the other hand, previous studies found that in IMS systems using NH_3_ as dopant and at temperatures lower than 50 °C, as in the case of the LCD-3.2E instrument used by us in this work, the monomer and dimer product ions are not protonated, but ammoniated species; this was demonstrated using mass spectrometry for product ions from DMMP (dimethyl methyl phosphonate), a known surrogate of nerve agents [39]; in conclusion, it may confidently be assumed that both product ions generated by CPF were, most probably, the ammoniated species CPF·NH_4_^+^ and CPF_2_·NH_4_^+^, respectively.

Concerning the figures of merit, LOD and LOQ obtained for Chlorpyrifos using the handheld ToF IMS instrument model LCD-3.2E (with ammonia doping, corona discharge ionization source and operated near ambient temperature) are very close to those related to the organophosphorus compound DIMP (diisopropyl methylphosphonate), which is simultaneously a surrogate compound for nerve chemical warfare agents Sarin and Soman and a precursor of Sarin; if in the case of DIMP the LOD and LOQ were found to be 0.24 ppb_v_ and 0.80 ppb_v_ (1.8 and 6 μg m^−3^) [40], for Chlorpyrifos (this work) LOD and LOQ were calculated as 0.72 ppb_v_ and 2.41 ppb_v_ (10.5 and 35.1 μg m^−3^), respectively.

## 5. Conclusions

This paper comes to report, for the first time, the successful IMS detection and quantification of CPF vapors in positive ion mode and in real time (seconds), using a handheld ToF IMS instrument operated near ambient temperature. Characteristic ion mobility spectra that included two product ion peaks were obtained. Reduced ion mobilities were determined using 2,4-lutidine as an ion mobility standard and were found to be as follows: K_0_ = ca. 1.76 cm^2^ V^−1^ s^−1^ (which may be assigned to the ammoniated monomer product ion) and K_0_ = ca. 1.47 cm^2^ V^−1^ s^−1^ (the dimer product ion). The identification of CPF by IMS is therefore reliably feasible using simultaneously both product ion peaks observed in the ion mobility spectrum.

The detection limit for CPF vapors was determined as LOD = 0.72 ppb_v_ (10.5 μg m^−3^), and the limit of quantification was LOQ = 2.41 ppb_v_ (35.1 μg m^−3^) CPF. Saturation of IMS response is thought to develop above 1000 ppb_v_ (14.6 mg m^−3^) CPF.

Through this study, it was also successfully proven that rapid sensing and quantifying vapors of CPF at ultra-trace levels is perfectly feasible using a pocket-held ToF IMS instrument with a non-radioactive (corona discharge) ionization source and operated near ambient temperature. This provides a series of strategic advantages—like excellent sensitivity, possibility for on-field analysis, low cost per analysis and real-time response—compared with many other analytical techniques.

Potential shortcoming of this work may be, most probably, related to potential interferences coming from other OP pesticides present in the air sample interrogated by this handheld ToF IMS instrument. However, a different OP molecule will generate product ions having different shapes (and hence reduced ion mobilities) than ions produced by CPF.

Finally, the main conclusion is that fast detection of vapors of CPF organophosphorus pesticide in the field, at ultra-trace levels (<1 ppm_v_), can be achieved quickly using handheld, highly integrated IMS instruments operated near ambient temperature, as is the case with the Lightweight Chemical Detector model LCD-3.2E. Furthermore, the applicability of this IMS-based method can successfully cover, for instance, rapid environmental research (for example, studies on CPF persistence), but also forensic or industrial hygiene investigations. Besides the mentioned applications, rapid trace detection of CPF vapors could probably be useful in studying and assessing the behavior of utterly toxic nerve chemical warfare agents, making it therefore a useful tool for research and training related to chemical weapons exposure.

## Figures and Tables

**Table 1:** Main properties of OP pesticide Chlorpyrifos (O,O-diethyl O-(3,5,6-trichloro-2-pyridyl) phosphorothioate) (adapted information from ref. [5]).

Substance Name and Formula	Properties
Chlorpyrifos O,O-diethyl O-(3,5,6-trichloro-2-pyridyl) phosphorothioate C_9_H_11_Cl_3_NO_3_PS CAS#: 2921-88-2 EC#: 220-864-4	Molecular mass: 350.57 g mol^−1^ Melting point: ca. 41…42 °C Boiling point: decomposes at approx. 160 °C Physical state: solid, white/colorless crystals Density: 1.4 g cm^−3^ (@ 20 °C) Relative density of vapors: 12.1 (air = 1) Vapor pressure: ca. 2·10^−5^ Torr @ 25 °C Volatility: 0.38 mg m^−3^ @ 25 °C Octanol–water partition coefficient (log K_ow_): 4.9…5.2 Solubility in water: low—ca. 1.4 mg L^−1^ (@ 25 °C) Soluble in organic solvents (acetone, benzene, carbon disulfide, etc.) Acute toxicity: medium to high, with LD_50_ of ca. 150 mg kg^−1^ (rat, oral), ca. 500…1000 mg kg^−1^ (goats, oral), ca. 1200 mg kg^−1^ (rabbit, dermal) and LC_50_ of ca. 550 mg m^−3^ (rat, inhalation, 4 h) Minimum risk level: 0.003 mg kg^−1^ day^−1^ (acute, oral) and 0.001 mg kg^−1^ day^−1^ (chronic, oral) Reported fatal dose: 300 mg kg^−1^ (human, adult) Occupational air level (OSHA): 0.04 mg m^−3^ (8 h) TWA: 0.2 mg m^−3^ (UK); STEL: 0.6 mg m^−3^ (UK) NIOSH recommended exposure limit (REL): 0.2 mg m^−3^ (10 h, skin); 0.6 mg m^−3^ (15 min, skin) TLV (threshold limit value)–TWA: 0.1 mg m^−3^ (8 h; inhalable fraction and vapor), skin EPA limit in air: 1 μg m^−3^ (adults) and 0.5 μg m^−3^ (children) Estimated AVDI (average daily food intake) in the U.S.: 0.04·10^−3^ mg day^−1^ in 1980; 0.8·10^−3^ mg day^−1^ in 1990. Conversion: 1 ppm_v_ = 14.57 mg m^−3^ (20 °C)

**Table 2:** Concentrations of CPF vapors in the standard atmospheres, correlated to volumes of injected standard reference material (with 2 ng μL^−1^ CPF) (for vapor concentrations < 100 ppb_v_), respectively, to volumes of injected standard solution (with 0.5 μg μL^−1^ CPF) obtained from Nurelle-D commercial product: C_CPF vapors_ = (V_standard sol._ × C_standard sol._)/V_jar_ (1 ppb_v_ = 14.57 μg m^−3^).

V_standard sol.−SRM_ [μL]	C_CPF_ [μg m^−3^]	C_CPF_ [ppb_v_]
8	22	1.5
16	44	3
32	89	6
63	175	12
132	366	25
265	736	50
**V_standard sol.−Nurelle D_** **[μL]**	**C_CPF_ [μg m^−3^]**	**C_CPF_ [ppb_v_]**
2.1	1458	100
7.4	5140	350
16.8	11,667	800

**Table 3:** Summary of experimental results obtained with CPF vapors using the LCD-3.2E handheld ToF IMS instrument in positive ion mode (in order to calculate standard deviation, three replicates were used for peak height). Notes: Peak of positive reactant ion (RIP) has t_d_ = 4.74 ms and its height is h_max_ = 8000 a.u. T_IMS_ = 301.5 K and P_IMS_ = 971.5 mbar = 729 mm Hg.

C_CPF_ [ppb_v_]	Drift Time Monomer t_d_ [ms]	Peak Height Monomer h_max_ [a.u.]	Drift Time Dimer t_d_ [ms]	Peak Height Dimer h_max_ [a.u.]
1.5	6.14	85 ± 6	-	-
3	6.14	130 ± 9	-	-
6	6.14	230 ± 14	-	-
12	6.16	500 ± 26	-	-
25	6.14	990 ± 58	-	-
50	6.16	1640 ± 84	-	-
100	6.06	2650 ± 152	7.28	290 ± 16
350	6.06	3540 ± 194	7.28	890 ± 48
800	6.06	3160 ± 168	7.26	1750 ± 92

**Table 4:** Reduced ion mobilities K_0_ calculated for ions produced by CPF vapors.

Operation Mode	Ion Drift Time, t_d_ [ms]		Reduced Ion Mobility ^1^, K_0_ [cm^2^ V^−1^ s^−1^]	Reduced Ion Mobility ^2^, K_0_ [cm^2^ V^−1^ s^−1^]
	Pos RIP:	4.74	2.278	2.251
Positive	PIP 1 (monomer):	6.06	1.783	1.760
	PIP 2 (dimer):	7.28	1.484	1.465

where ^1^—Calculated by the IMS software TrimScan. ^2^—Calculated using the IMS cell constant (A): K_0_ = (A/t_d_). PIP—product ion peak; RIP—reactant ion peak.

**Table 5:** Resolving power of the LCD-3.2E IMS instrument for CPF (spectrum for 100 ppb_v_).

Ion Drift Time, t_d_ [ms]		Peak Width at Half Maximum, Δt_d_ [ms]	Resolving Power, R_IMS_
Pos RIP:	4.74	0.35	13.5
PIP 1 (monomer):	6.06	0.36	16.8
PIP 2 (dimer):	7.28	0.42	17.3

**Table 6:** Detection and quantification of CPF using various IMS and hyphenated GC-IMS systems. RS—IMS instrument equipped with a radioactive ionization source; NRS—IMS instrument equipped with a non-radioactive ionization source.

Application/Instrument Used	K_0_ [cm^2^ V^−1^ s^−1^]	Quant.	Ref.
*Determination of CPF residue in pistachio oil after liquid extraction by IMS:* Home-built IMS (Isfahan Univ. of Technology) with corona discharge ionization (NRS). Drift length l_d_ = 11 cm. E = 500 V cm^−1^. T_IMS cell_ = 190 °C. Drift gas: N_2_. Extraction of CPF from pistachio oil was performed with hexane. Liquid extract was injected into the IMS at 220 °C. Linear dynamic range LDR: 0.4 to 20 μg g^−1^.	Only one product ion peak was noticed, at t_d_ = ca. 11 ms (POS mode). K_0_ not reported!	LOD 0.1 μg g^−1^ LOQ 0.3 μg g^−1^	[17]
*Determination of CPF in water and food samples by tandem GC-IMS:* CPF was extracted by SPME from various matrices (fruits, vegetables, water), and then, analysis by GC—IMS was performed. NRS IMS (corona discharge) was used. T_IMS cell_ = 200 °C. Drift length l_d_ = 11 cm (?). E = 500 V cm^−1^. Drift gas: N_2_. Linear dynamic range LDR: 0.50 to 25 μg L^−1^.	Only one product ion peak was observed (t_d_ = ca. 11.2 ms), overlapping with that produced by Trifluralin, at t_d_ ca. 11.4 ms (POS mode). K_0_ not reported!	LOD 0.15 μg L^−1^ LOQ 0.50 μg L^−1^	[18]
*Determination of CPF after microextraction using Cu COF by IMS:* Samples: water, soil, pear fruits. IMS model IMS 300 (TOF Tech., Iran) with NRS (corona discharge). T_IMS cell_ = 200 °C. Drift length l_d_ = 11 cm (?). E = 500 V cm^−1^ (?). Linear dynamic range LDR: 1.0 to 400.0 ng mL^−1^.	Only one product ion peak was noticed, at t_d_ = ca. 11.7 ms (POS mode). K_0_ not reported!	LOD 0.65 ng mL^−1^	[19]
*Determination of CPF after ultrasound-assisted microextraction using IMS:* Samples: water, food (vegetables, fruits). IMS model IMS 400 (TOF Tech. Pars Co., Iran) with NRS (corona discharge). Drift length l_d_ = 11 cm; drift gas: dry air. T_IMS cell_ = 200 °C. E = 500 V cm^−1^. Linear dynamic range LDR: 5.0 to 200.0 μg L^−1^.	Only one product ion peak was noticed (POS mode), at t_d_ = ca. 10.3 ms. K_0_ not reported!	LOD 1.3 μg L^−1^	[20]
*Determination of CPF after headspace SPME by IMS:* Various aqueous samples (river, farm, groundwater) and fruit juices. IMS model CD-1400 (TOF Tech. Pars Co., Isfahan, Iran) with NRS (corona discharge). Drift length l_d_ = 11 cm; drift gas: nitrogen. T_IMS cell_ = 190 °C. E = 500 V cm^−1^ (?). Linear dynamic range LDR: 0.5 to 300.0 ng mL^−1^.	Only one product ion peak was noticed, at t_d_ = ca. 9.3 ms (POS mode). K_0_ not reported!	LOD 0.2 ng mL^−1^	[21]
*Determination of CPF after DLLME by IMS:* Samples of water, fruits (apples), vegetables (tomatoes). IMS (Teif Azmon Espadana Co., Isfahan, Iran) with NRS (corona discharge). Drift length l_d_ = 11 cm; drift gas: nitrogen. T_IMS cell_ = 160 °C. E = 450 V cm^−1^. Linear dynamic range LDR: 0.1 to 3.0 μg L^−1^.	Only one product ion peak was reported, at t_d_ = ca. 10.9 ms (and at 12.2 ms in spiked samples!) (POS mode). K_0_ not reported!	LOD 0.04 μg L^−1^	[22]
*Determination of CPF after SPME by IMS:* Sample: standard solution of CPF. IMS (Teif Azmon Espadana Co., Isfahan, Iran) with NRS (corona discharge). Drift length l_d_ = 11 cm; drift gas: nitrogen. T_IMS cell_ = 160 °C. E= 420 V cm^−1^. Linear dynamic range LDR: 0.5 to 20.0 μg L^−1^.	Only one product ion peak was noticed, at t_d_ = ca. 12.4 ms (POS mode). K_0_ not reported!	LOD 0.15 μg L^−1^ LOQ 0.5 μg L^−1^	[23]
*Determination of CPF after ultrasound-assisted emulsification microextraction using IMS:* Samples: rice paddy water, rice. Home-built IMS with RS (^63^Ni). Drift length l_d_ = 10 cm; drift gas: nitrogen. T_IMS cell_ = 200 °C. E = 550 V cm^−1^. Linear dynamic range LDR: 8.9 to 750.0 μg L^−1^.	Only one product ion peak was reported, at t_d_ = ca. 11.5 ms (POS mode). K_0_ not reported!	LOD 3.2 μg L^−1^	[24]
*Determination of CPF after SPME by IMS:* Samples: water, fruits (grape, tangerine). IMS (Teif Azmon Espadana Co., Isfahan Univ. of Technology, Iran) with NRS (corona discharge). Drift length l_d_ = 11 cm; drift gas: nitrogen. T_IMS cell_ = 160 °C. E = 400 V cm^−1^. Linear dynamic range LDR: 0.1 to 10.0 μg L^−1^.	Only one product ion peak was noticed, at t_d_ = ca. 11.4 ms (POS mode). K_0_ not reported!	LOD 0.05 μg L^−1^ LOQ 0.10 μg L^−1^	[25]
*Determination of CPF after SPME by IMS:* Samples: water, fruit juices. IMS model CD-1400 (Theif Azmoon Espadana Co., Isfahan, Iran) with NRS (corona discharge). Drift length l_d_ = 11 cm; drift gas: nitrogen. T_IMS cell_ = 200 °C. E = 500 V cm^−1^ (?). Linear dynamic range LDR: 2 to 250.0 ng mL^−1^.	Only one product ion peak was noticed, at t_d_ = ca. 9.2 ms (POS mode). K_0_ not reported!	LOD 0.6 ng mL^−1^	[26]
*Determination of CPF by tandem GC-IMS after SPME:* CPF was extracted by dispersive SPME from various matrices (water, fruits, vegetables), and then, analysis by GC—IMS was performed. IMS manufactured by Teif Azmon Espadana Co., Isfahan, Iran, with NRS (corona discharge) was used. Drift length l_d_ = 11 cm. T_IMS cell_ = 200 °C. Drift gas: N_2_. E = 500 V cm^−1^. Linear dynamic range LDR: 2 to 1000 μg L^−1^.	Only one product ion peak was observed (t_d_ = ca. 11.8 ms), overlapped with peak produced by Malathion (ca. 12.0 ms).—POS mode. K_0_ not reported!	LOD 0.85 μg L^−1^ LOQ 2 μg L^−1^	[27]
*Determination of CPF after SPME by tandem GC-IMS:* CPF was extracted by SPME from various matrices (water, fruits, vegetables), and then, analysis by GC—IMS was performed. Home-built IMS (Isfahan Univ., Iran), with NRS (corona discharge) used. Drift length l_d_ = 11 cm; drift gas: nitrogen. T_IMS cell_ = 230 °C. E = 500 V cm^−1^. Linear dynamic range LDR: 0.025 to 2.0 μg L^−1^ (river water, wastewater); 0.75 to 20.0 μg kg^−1^ (pears, grapes); 0.50 to 15.0 μg kg^−1^ (eggplants).	Only one product ion peak was observed, with t_d_ = ca. 14.1 ms (POS mode). K_0_ not reported!	LOD 0.010 μg L^−1^ LOQ 0.025 μg L^−1^ (water) LOD 0.30 μg L^−1^ LOQ 0.75 μg L^−1^ (pears, grapes)	[28]
*Determination of CPF after SPME by tandem GC-IMS:* CPF was extracted by SPME from various matrices (water, milk, serum, fruits), and then, analysis by GC—IMS was performed. Home-built IMS (Isfahan Univ. of Technology, Iran), with NRS (corona discharge). Drift length l_d_ = 11 cm; drift gas: N_2_. T_IMS cell_ = 200 °C. E = 500 V cm^−1^. Linear dynamic range LDR: 0.05 to 20.0 μg L^−1^ (water).	No IMS spectrum was provided. K_0_ not reported!	LOD 0.019 μg L^−1^ (water) LOQ 0.050 μg L^−1^ (water)	[29]
*Determination of CPF after liquid phase micro-extraction by IMS:* Samples: water, vegetables. IMS (Teif Azmon Espadana Co., Isfahan, Iran) with NRS (secondary electrospray ionization SESI). Drift length l_d_ = 11 cm; drift gas: N_2_. T_IMS cell_ = 150 °C. E = 567 V cm^−1^. Linear dynamic range LDR: 1 to 70.0 μg L^−1^.	Only one product ion peak was noticed, at t_d_ = 12.28 ms (POS mode). **K_0_ = 1.37 cm^2^ V^−1^ s^−1^** (with nicotinamide as mobility standard)	LOD 0.21 μg L^−1^ LOQ 0.70 μg L^−1^	[30]
*Determination of CPF after micro-extraction by IMS:* Sample: water. Home-built IMS (Isfahan Univ., Iran), with NRS (corona discharge) was used. Drift length l_d_ = 11 cm; drift gas: N_2_. T_IMS cell_ = 150 °C. E = 400 V cm^−1^. Linear dynamic range LDR: 2 to 200 μg L^−1^.	Only one product ion peak was noticed, at t_d_ = ca. 10.6 ms (POS mode). K_0_ not reported!	LOD 0.6 μg L^−1^ LOQ 2.0 μg L^−1^	[31]
*Determination of CPF after SPME by tandem GC-IMS:* Sample: water. Home-built GC-IMS (Isfahan Univ., Iran), with NRS (corona discharge) was used. Drift gas: N_2_. T_IMS cell_ = 235 °C. E = 500 V cm^−1^. Linear dynamic range LDR: 0.02 to 5 μg L^−1^.	Only one product ion peak was noticed, at t_d_ = ca. 12.1 ms (POS mode). **K_0_ = 1.27 cm^2^ V^−1^ s^−1^** (with nicotinamide as mobility standard).	LOD 0.012 μg L^−1^	[32]
*Determination of CPF after dispersive solid-phase extraction by IMS:* Samples: water, vegetables. Home-built IMS model 1000 (Isfahan Univ. of Technology, Iran), with NRS (corona discharge) was used. Drift length l_d_ = 16 cm; drift gas: N_2_. T_IMS cell_ = 200 °C. E = 500 V cm^−1^. Reactant ion: NH_4_^+^. Linear dynamic range LDR: 1 to 500 ng mL^−1^.	Only one product ion peak was noticed, at t_d_ = ca. 8.8 ms. K_0_ not reported!	LOD 0.3 ng mL^−1^ LOQ 1 ng mL^−1^	[33]
*Determination of CPF after dispersive solid-phase extraction by IMS:* Samples: water, fruit juice, vegetables. IMS model CD-1400 (TOF Tech, Isfahan, Iran), with NRS (corona discharge), was used. Drift length l_d_ = 11 cm; drift gas: N_2_. T_IMS cell_ = 200 °C. E = 636 V cm^−1^. Linear dynamic range LDR: 0.6 to 300.0 ng mL^−1^.	Only one product ion peak was noticed, at t_d_ = ca. 11 ms (POS mode). K_0_ not reported!	LOD 0.2 ng mL^−1^	[34]
*Determination of CPF after dispersive liquid–liquid microextraction by IMS:* Samples: water, vegetables (potato). IMS model CD-1400 (Teif Azmon Espadana Co., Isfahan, Iran), with NRS (corona discharge) was used. Drift length l_d_ = 11 cm; electric field E = 420 V cm^−1^; drift gas: N_2_. T_IMS cell_ = 160 °C. Linear dynamic range LDR: 0.1 to 7.0 μg L^−1^.	Only one product ion peak was noticed, at t_d_ = ca. 13.2 ms (POS mode). K_0_ not reported!	LOD 0.03 μg L^−1^ LOQ 0.1 μg L^−1^	[35]
*Determination of CPF after stir mesh screen sorptive extraction (SMSE) by IMS:* Samples: water (river, well, agricultural wastewater); fruits (apple). IMS with thermal desorption unit (Teif Azmon Espadana Co., Isfahan Univ. of Technology, Iran), with NRS (corona discharge) was used. Drift length l_d_ = 11 cm; electric field E = 500 V cm^−1^; drift gas: nitrogen. T_IMS cell_ = 150 °C. Linear dynamic range LDR: 0.1 to 20.0 μg L^−1^.	Only one product ion peak was noticed, at t_d_ = ca. 11.3 ms (POS mode). K_0_ not reported!	LOD 0.035 μg L^−1^ LOQ 0.100 μg L^−1^	[36]
*Determination of CPF after thin film micro-extraction (SMSE) by IMS:* Samples: water (well, river, agricultural wastewater); fruits (tangerine). IMS with thermal desorption unit (Isfahan Univ. of Technology, Iran), with NRS (corona discharge) was used. Drift length l_d_ = 11 cm; electric field E = 450 V cm^−1^; drift gas: N_2_. T_IMS cell_ = 160 °C. Linear dynamic range LDR: 1 to 100 μg L^−1^.	Only one product ion peak was noticed, at t_d_ = ca. 12.8 ms (POS mode). K_0_ not reported!	LOD 0.3 μg L^−1^	[37]
*Indoor and outdoor determination of pesticides in air after active sampling on Teflon membranes by IMS:* Samples: air. Commercial IMS with RS (^63^Ni) with thermal desorption, model Ionscan-LS (Smiths Detection, Morristown, NJ, USA). Drift Tube length: 7 cm; electric field E = 252 V cm^−1^; drift gas: dry air. T_IMS cell_ = 237 °C. Linear dynamic range LDR—over 2 to 10 ng.	One product ion peak in the Negative ion mode, at t_d_ = 11.93 ms. **K_0_ = 1.561 cm^2^ V^−1^ s^−1^** (using 4-nitrobenzonitrile as mobility standard, with K_0_ = 1.655 cm^2^ V^−1^ s^−1^).	LOD 600 pg LOQ 1960 pg	[38]
*Determination of CPF vapors by IMS:* ToF IMS, model LCD-3.2E (NRS—corona discharge), operated near ambient T (ca. 28 °C); ammonia doped. Calibration: from 1.5 to 800 ppb_v_ Linear range: from 2.5 to 100 ppb_v_ Saturation: >1000 ppb_v_	1.727 (monomer) 1.461 (dimer)	LOD 0.72 ppb_v_ LOQ 2.41 ppb_v_	this work

Note: LOD is the limit of detection and LOQ the limit of quantification, respectively.

## Data Availability

The original contributions presented in the study are included in the article; further inquiries can be directed to the corresponding authors.
